# Enhanced Extracellular Matrix Breakdown Characterizes the Early Distraction Phase of Canine Knee Joint Distraction

**DOI:** 10.1177/19476035211014595

**Published:** 2021-05-20

**Authors:** Michelle Teunissen, Alberto Miranda Bedate, Katja Coeleveld, Frank M. Riemers, Björn P. Meij, Floris P. J. G. Lafeber, Marianna A. Tryfonidou, Simon C. Mastbergen

**Affiliations:** 1 Department of Clinical Sciences, Faculty of Veterinary Medicine, Utrecht University, Utrecht, The Netherlands; 2 Rheumatology & Clinical Immunology, UMC Utrecht, Utrecht University, Utrecht, The Netherlands

**Keywords:** cartilage regeneration, groove model, osteoarthritis

## Abstract

**Objective:**

Joint distraction triggers intrinsic cartilage repair in animal models of osteoarthritis (OA), corroborating observations in human OA patients treated with joint distraction. The present study explores the still largely elusive mechanism initiating this repair process.

**Design:**

Unilateral OA was induced in the knee joint of 8 dogs using the groove model; the contralateral joint served as a control. After 10 weeks, 4 animals received joint distraction, the other 4 serving as OA controls. Halfway the distraction period (after 4 weeks of a standard 8-week distraction treatment), all animals were euthanized, and joint tissues were collected. A targeted quantitative reverse transcription polymerase chain reaction (qRT-PCR) analysis was performed of commonly involved processes including matrix catabolism/anabolism, inflammation, and known signaling pathways in OA. In addition, cartilage changes were determined on tissue sections using the canine OARSI (Osteoarthritis Research Society International) histopathology score and collagen type II (COL2A1) immunostaining.

**Results:**

Midway distraction, the distracted OA joint showed an upregulation of proteolytic genes, for example, *ADAMTS5*, *MMP9*, *MMP13*, compared to OA alone and the healthy joints, which correlated with an increased OARSI score. Additionally, genes of the transforming growth factor (TGF)-β and Notch pathway, and markers associated with progenitor cells were increased.

**Conclusions:**

Joint distraction initiates both catabolic and anabolic transcriptional responses. The enhanced turnover, and thereby renewal of the matrix, could be the key to the cartilage repair observed in the months after joint distraction.

## Introduction

Osteoarthritis (OA) is a progressive joint disease characterized by inflammation and structural changes of the joint, causing pain and functional disability. The prevalence of knee OA approaches 5% of the global population, and is expected to rise due to increased age and prevalence of obesity of the population.^1-3^ This will significantly affect societal health and economic costs. For patients with significant joint damage and severe OA symptoms despite conservative therapy, knee arthroplasty is considered an effective therapy. However, when patients are relatively young (<60 years of age), the prostheses’ limited life span brings a greater risk of a future revision surgery.^4,5^ Therefore, there is a need for alternative treatment strategies that can delay, or even prevent, knee arthroplasty. Within this context, joint distraction has been proposed as a joint preserving treatment strategy. During joint distraction, the 2 bony ends of a joint are temporarily (6-9 weeks) distracted using an external fixation frame. The clinical application and efficacy of knee joint distraction (KJD) have been reviewed,^6-10^ and although the number of studies is limited and the sample size relatively small, there is evidence for a prolonged clinical benefit.^10-14^

At the biological level, there are indications that joint distraction facilitates cartilage regenerative effects. This reparative activity is evaluated in clinical studies by surrogate markers such as imaging and (serum/urine) biochemical markers. The strongest tissue repair is observed at 1 and 2 years after distraction by radiographic and magnetic resonance imaging evaluation (both quantitative and qualitative, such as dGEMRIC and T2 relaxation), the latter demonstrating the presence of hyaline cartilage.^11,12,15^ In addition, increased serum levels of the collagen type II synthesis marker, PIIANP (N-propeptide of collagen IIA), and decreased levels of the collagen type II breakdown marker, CTXII (C-Telopeptide of type II collagen), at 1 and 2 years post distraction treatment, show an increased ratio of synthesis over breakdown in this period.^11,16^ After this period, the degenerative, progressive nature of OA takes over again, and this results in a gradual waning of the effect. However, especially considering the natural progression in case of only conservative treatment, there is still improvement after 5 to 10 years compared to pretreatment situation.^13,14^ Complementary, a canine OA model, in which OA was induced in a period of 10 weeks using the groove model,^17^ followed by 8 weeks of KJD, showed structural improvement of the cartilage at 25 weeks follow-up after distraction.^18^ This experimental study showed that OA-knee joints treated with KJD had improved macroscopic and histopathology OARSI scores (the canine Osteoarthritis Research Society International [OARSI] assessment system),^19^ higher proteoglycan (PG) content, better retention of newly formed PG and less collagen damage compared to the OA control knee joints after the same prolonged follow-up.^18^

Altogether, the aforementioned findings support the notion that KJD elicits a reparative response, but the underlying mechanisms of action during distraction remain elusive. Involvement of multiple mechanisms have been postulated, including the temporary absence of mechanical loading while preserving joint fluid pressure oscillation, enhanced periarticular bone turnover, and/or stem cell modulation as a result of joint distraction.^11,20-23^ In order to further strengthen these existing hypotheses, the present study explored the initial transcriptional response of the cartilage and adjacent joint tissues during KJD treatment in the groove model, a canine OA model. More specifically, genes related to cartilage matrix turnover and bone remodeling, (cartilage) progenitor cell markers, cytokines, and signaling pathways involved in OA were investigated. As the first (bio)molecular changes are thought to start already during the treatment with joint distraction, a time point halfway (4 weeks) the common 8-week distraction period was selected for the analysis.

## Methods

### Animal Procedures

Skeletally mature Mongrel dogs (*n* = 8 females, mean ± standard deviation [SD] age 29 ± 7.6 months, mean weight 23 ± 2.8 kg) were used. Upon ethical approval by the local ethics committee on animal experimentation (2013.III.08.054), OA was induced in all dogs unilaterally (the right knee joint) according to the groove model.^17^ Grooves were applied using a Kirschner-wire (1.5-mm diameter) bent 0.4 mm from the top at 90° to ensure that the depth of the grooves was restricted to the cartilage depth and not to the subchondral bone. The contralateral, left knee joint served as healthy control (control) without further treatment. Ten weeks post OA induction, dogs were randomly divided into 2 groups of 4 animals. One group received no additional treatment (OA), while the other group received KJD (distraction). Briefly, to initiate KJD, bone pins were drilled into the femur and tibia and connected to external fixation frames in a 3-point fixation with the use of commercially available connectors. Subsequently, the external fixation frames on the femur and the tibia were connected by hinges medially and laterally of the knee joint. Distraction of the joint was carried out by extending the connecting rods and was visualized by fluoroscopy using a C-arm, while smooth motion of the joint during flexion and extension was maintained. Halfway the commonly used distraction period of 8 weeks, thus after 4 weeks of joint distraction, all 8 animals were euthanized, and material was collected for further analysis. A full description of experimental procedures can be found in the Supplementary Material.

### Collection of Material Postmortem and Tissue Processing

Within 1 hour after euthanasia, high-resolution photographs of the joint surfaces were obtained for macroscopic grading of cartilage damage after which tissues where processed. Cartilage tissue, excluding any subchondral bone, was collected from the weight-bearing area of the femoral condyles and tibial plateaus and processed for 2 purposes: fixed in 4% phosphate-buffered formalin containing 2% sucrose (pH 7.0) for (immuno)histochemistry, and snap frozen for RNA isolation. Additionally, tissue samples of fat pad, suprapatellar synovium, meniscus, and tibial and femoral subchondral bone samples were collected and snap frozen for RNA isolation.

### Cartilage Quality Assessment and Immunohistochemistry

Cartilage damage was macroscopically graded (2 observers; FPL, SCM) and microscopically graded on Safranin-O/Fast green stained sections (3 observers; SCM, MAT, and AMB) according to the OARSI canine scoring system.^19^ All samples were randomized and observers were blinded for the source of material studied. Data are provided as mean OARSI score ± SD. Furthermore, immunopositivity for collagen type-1 (COL1A1), -2 (COL2A1), and -10 (COLX) of the cartilage matrix was evaluated (Supplementary Material).

### Transcriptional Profiling

Tissue samples were reduced to powder (cartilage, meniscus, subchondral bone) and/or submitted to a short Tissuelyser (Qiagen) cycle (25 shakes/second for 4 minutes; fat pad, suprapatellar synovium). Thereafter, total RNA was extracted using the miRCURY RNA Isolation Kit (Exiqon, Vedbaek, Denmark) for cartilage and the RNeasy Mini Kit (Qiagen, Venlo, the Netherlands) for the other tissues, according to the manufacturer’s instructions with an additional on column DNase treatment (Qiagen). RNA quality and quantity were measured with a Bioanalyzer (Nano-chip, Agilent Technologies, Amstelveen, the Netherlands). cDNA was produced using the iScriptTM cDNA Synthesis Kit (Bio-Rad, Veenendaal, the Netherlands) with a similar RNA input for all samples following manufacturer’s instructions.

Total RNA was profiled with a panel of 63 genes by quantitative reverse transcriptase polymerase chain reaction (qRT-PCR; Supplementary Material). Investigated targets and/or pathways included genes related to cartilage matrix metabolism, bone remodeling, the TGF/BMP pathway,^24^ the IGF pathway,^25^ the Notch pathway,^26^ the Wnt pathway,^27^ the Indian Hedgehog pathway,^27^ and several cytokines, and progenitor cell associated markers.^28,29^ Based on initial analysis, additional target genes (*n* = 13) within these pathways were studied specifically for the cartilage tissue. Quantitative PCR was performed using a CFX384 Touch Real-Time PCR Detection System and IQ SYBR Green SuperMix (both from Biorad) according to the manufacturer’s protocols. Standard curves consisted of 4-fold serial dilutions of the cDNA template. For each standard curve, the amplification efficiency was between 90% and 110%. The normalization of gene expression was performed with 7 reference genes: *RPS19*, *Sdha*, *YWHAZ*, *TBP*, *RPS5*, *RPL13*, and *HPRT*.

### Statistical Analysis

ΔCt values, and OARSI histopathology scores, were statistically analyzed (R version 3.6.3,^30^ RStudio version 1.2.5033^31^) for the 3 comparison groups (OA vs. control, distraction vs. OA, and distraction vs. control). Linear models were employed for the analysis of variance (ANOVA). For each parameter, the selection of random effects for the different linear models was performed, considering variables “donor” and “location” (tibial plateaus/femoral condyles [if applicable depending on the tissue]). If the variable “location” was considered a significant variable for the model, an additional analysis was run on the separated data of the tibial plateaus and femoral condyles of the cartilage and subchondral bone. Normality of the residuals, homoscedasticity, independence of errors, and the presence of outliers were assessed for each linear model. If any of the assumptions were not held, a power transformation of the dCt values with the *lambda* coefficient as exponent was performed, reassessing all the assumptions. If the assumptions were not passed, an exact Wilcoxon-Mann-Whitney test, which is a permutation based nonparametric test, was used. *P* values were subjected to corrections for multiple testing (Benjamini-Hochberg false discovery rate). Effect sizes (ES) and ES’s 95% confident intervals (CI) were provided as Hedge’s *g* for normally distributed data and Cliff’s delta for nonnormally distributed data. In the event a specific gene could not be detected in 1 of the 2 groups, this was considered a biologically relevant difference in expression between groups. These comparisons were included as significant with a fold change >10. Specific details of the statistical analysis can be found in the Supplementary Material.

## Results

### Cartilage Integrity Is Still Deteriorated Midterm Distraction Treatment

**Figure 1.**
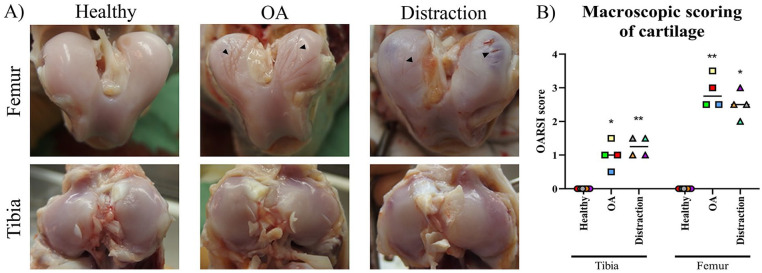
Macroscopic cartilage damage assessment at midterm (4 weeks) of the distraction treatment. (**A**) Macroscopic photographs of canine cartilage after 4 weeks of the distraction for tibial plateaus (Tibia) and femoral condyles (Femur) in the experimental groups: control cartilage (Healthy), OA cartilage (OA), and distracted OA cartilage (Distraction). The grooves that were surgically applied were still visible (black arrowheads) with additional surrounding degeneration, while the condylar cartilage of the contralateral control knees was intact. (**B**) OARSI scoring of macroscopic cartilage after 4 weeks of distraction for femoral condyles (Femur) and tibial plateaus (Tibia) in control cartilage (Healthy), OA and OA distracted cartilage (Distraction). “*Y*” axes represent OARSI grade and “*X*” axes the experimental conditions. Asterisks indicate statistically significant differences compared to the healthy control (*P < 0.05; **P < 0.01).

**Figure 2.**
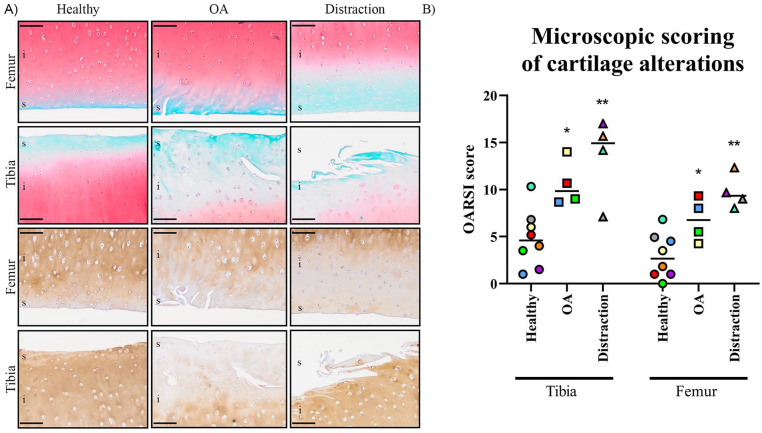
Representative immunostainings and OARSI grading of the cartilage at midterm (4 weeks) distraction treatment. (**A**) Representative images of Safranin-O/Fast Green staining and collagen type II immunohistochemistry from control (healthy), osteoarthritic (OA), and OA + KJD (Distraction) joints after 4 weeks of distraction of the tibial plateaus (tibia) and femoral condyles (femur). Scale bar = 100 µm. (S) = superficial layer; (I) = intermediate layer. (**B**) OARSI scoring of the histology after 4 weeks of distraction is given for all conditions, in cartilage from the femoral condyles and tibial plateaus. Asterisks indicate statistically significant differences between the indicated groups within the locations with at least medium effect sizes (*P < 0.05; **P < 0.01).

Fourteen weeks after OA induction, the OA group (without distraction) clearly showed macroscopic cartilage damage in comparison to the control (OARSI score: femur 2.9 ± 0.5 vs. 0.0 ± 0.0, *P* < 0.005; tibia 1.0 ± 0.4 vs. 0.0 ± 0.0, *P* < 0.015; **Fig. 1**). A similar degree of cartilage damage was found in the distraction group (femur 2.5 ± 0.4, *P* < 0.015; tibia 1.3 ± 0.3, *P* < 0.005 [vs. control]); OA and distraction groups did not differ (**Fig. 1**). These macroscopic observations were confirmed by histological analysis. The average histopathology OARSI score was significantly higher in the OA group compared to control joints (femur 6.8 ± 2.3 vs. 3.0 ± 2.4, *P* < 0.02; tibia 10.6 ± 2.4 vs. 4.8 ± 3.0, *P* < 0.02; **Fig. 2B**). The distraction group showed on average a higher histopathology OARSI score compared to the OA condition (femur 9.8 ± 1.9, *P* = 0.08, with a very large ES; tibia 13.5 ± 4.4, *P* = 0.2, with a large ES; **Fig. 2B**, Supplementary Material). COL1A1 and COLX proteins were undetectable in the cartilage tissues (Supplementary Material). A loss of COL2A1 staining into the intermediate cartilage layer was observed in the distraction and OA group (**Fig. 2A**).

### Gene Expression Profiling of the OA Cartilage and OA Subchondral Bone Shows Minimal Changes at the Transcriptional Level

**Figure 3.**
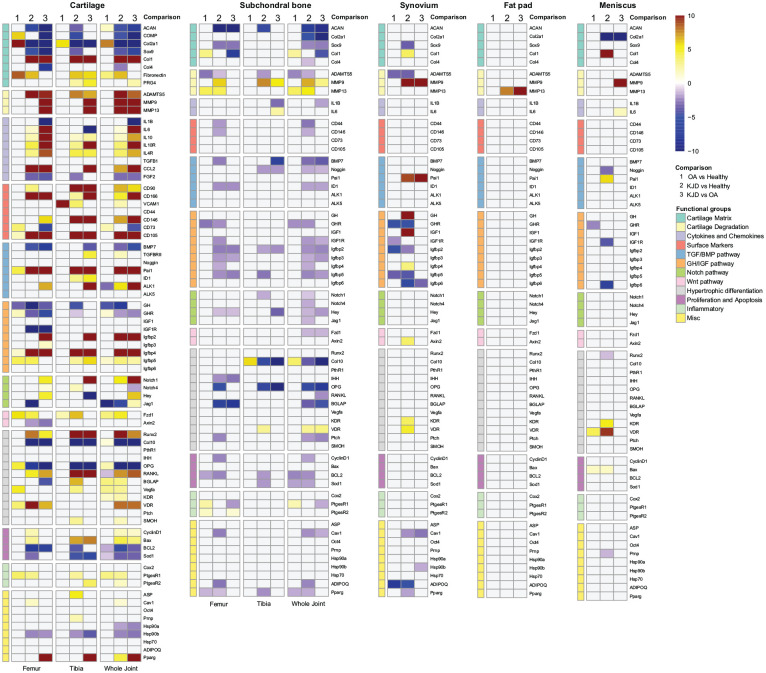
The transcriptional profile of cartilage, subchondral bone, synovium, meniscus, and fat pad. Cartilage and subchondral bone (Bone) registered the highest numbers of differentially expressed (DE-) genes from all analyzed tissues based on the corresponding DE-genes heat-map. Scale bars represent inverted, significant ΔΔCt values in a color gradient (dark blue [highly downregulated gene], white [no differences], and dark red [highly upregulated gene]). Not significant DE-genes are depicted in gray. For each tissue, the heat map is divided in comparisons and anatomical locations. For the cartilage and subchondral bone the following anatomical locations were used: Whole joint (no differentiation between anatomical locations), Tibia (tibial plateaus), Femur (femoral condyles). The comparisons depicted on top of the heat map include: (1) osteoarthritic (OA) compared to healthy joints (OA vs. healthy [green comparison]), (2) distracted joints (KJD) compared to healthy joints (KJD vs. healthy [orange comparison]), and (3) KJD compared to OA joints (KJD vs. OA [lavender comparison]). Furthermore, DE-genes are divided in functional groups, characterized at the left side of heat map by colored boxes.

Taken together, the most differentially expressed (DE) genes were detected in cartilage and subchondral bone (**Fig. 3**). Genes with at least 2-fold difference were considered biologically relevant to report and discuss.

Compared to the control joints, 25% (19/76) DE-genes were detected in the OA cartilage, of which 10 showed more than a 2-fold change (**Fig. 3**). Interestingly, the cartilage matrix gene *COL2A1* was upregulated in the OA cartilage (*COL2A1*, *P* < 0.001), while catabolic genes, generally associated with OA, Matrix Metalloproteinase 13 (*MMP13*), and A Disintegrin and Metalloproteinase with Thrombospondin Motifs 5 (*ADAMTS5*) did not differ significantly. Additionally, genes related to the hypertrophic differentiation *COLX*, Bone Gamma-Carboxyglutamate Protein (*BGLAP*), and Vascular Endothelial Growth Factor A (*VEGFA*) were significantly upregulated (*P* < 0.05).

In the subchondral bone, only 9% (9/63) of the genes were significantly different, of which 7 were more than 2-fold regulated (**Fig. 3**). Noteworthy is the upregulation of *MMP13* (*P* < 0.01), while *ADAMTS5* was downregulated in the bone (*P* < 0.001). Furthermore, *COLX* (*P* < 0.05) and *COL1A1* (*P* < 0.05), as well as the inflammatory marker Prostaglandin E Synthase (*PTGES)-1* (*P* < 0.05) were significantly upregulated.

In the synovium, 10% (6/63) of the genes were detected as DE-genes, all more than 2-fold different. Most of these genes were involved in the IGF pathway, including Growth Hormone Receptor (*GHR*), IGF 1 Receptor (*IGF1R*), and IGF Binding Proteins 2 and 5 (*IGFBP-2, -*5), and were significantly downregulated (*P* < 0.05) in OA. In addition, comparable with the bone, *ADAMTS5* was downregulated (*P* < 0.01). No significant DE-genes were found in the osteoarthritic fat pad and meniscus compared to the healthy joints.

### Anabolic and Catabolic Transcriptional Responses Coincide in the Distracted Cartilage

In the distracted cartilage 58% (44/76) DE-genes were detected compared to the OA cartilage, of which 42 showed more than a 2-fold change. A clear catabolic transcriptional response was observed; *MMP9*, *MMP13*, and *ADAMTS5* were highly upregulated in the distracted cartilage (*P* < 0.001) compared to OA. Furthermore, the cartilage matrix genes Aggrecan (*ACAN*), *COL2A1*, Cartilage Oligomeric Matrix Protein (*COMP*), and the master chondrogenic regulator SRY-Box Transcription Factor 9 (*SOX9*) were severely downregulated (*P* < 0.001), while *COL1A1*, a marker of fibrocartilage, was upregulated (*P* < 0.001) compared to OA cartilage. Interestingly, although RUNX Family Transcription Factor 2 (*RUNX2*, *P* < 0.001), a marker of chondrocyte hypertrophy, was upregulated, *COLX* was downregulated (*P* < 0.001).

At the same time, putative anabolic transcriptional responses were found, including genes in the Notch pathway (upregulation of *NOTCH1* [*P* < 0.001] and Hairy/enhancer-of-split related with YRPW motif protein 1 [*HEY1*], *P* < 0.05), and the TGF-β pathway (upregulation of activin receptor-like kinase-1 [*ALK1*]; *P* < 0.0001), plasminogen activator inhibitor 1 (*PAI1*; *P* < 0.0001) and the TGF-β receptor II (*TGFβRII*) specifically in the tibial plateaus (*P* < 0.05). Additionally, several cytokines were only expressed in distracted cartilage including interleukin 6 (*IL-*6), chemokine C-C motif ligand 2 (*CCL2*), and the anti-inflammatory cytokine *IL-10* and its receptor, *IL-10R*, while they were undetectable in OA cartilage. Furthermore, several markers associated with progenitor cells were upregulated such as the surface markers *CD105*, *CD90*, *CD166*, and *CD146* (all *P* < 0.001).

### Subchondral Bone of the Distracted Joined Showed Certain Coincidental Transcriptional Features with Cartilage

In the subchondral bone of the distracted joint, 26 (37%) DE-genes were found compared to the OA joint, of which 15 showed more than a 2-fold regulation. Noteworthy was the downregulation of *COL1A1* and *BGLAP* in the subchondral bone of the distracted joint compared to the OA joint (*P* < 0.001). Furthermore, in correspondence with the distracted cartilage, a downregulation of *ACAN* (*P* < 0.001) and *COL2A1* (*P* < 0.05) and an upregulation of *MMP9* (*P* < 0.01) was found.

### Changes in the Synovium, Fat Pad, and Meniscus of the Distracted Joint Are Limited and Coincide with the Distracted Cartilage and Subchondral Bone

In the synovium of the distracted joint, only 4 DE-genes were detected that showed more than a 2-fold change compared to the OA joint. A significant upregulation of *MMP9* (*P* < 0.05) and *PAI1* (*P* < 0.05) was found as well as a significant upregulation of *IGFBP6* (*P* < 0.05). In the distracted joint, *COL2A1* was significantly downregulated in the meniscus compared to the OA condition (*P* < 0.05), and *MMP9* and *IL6* were only detected in distracted cartilage, coinciding with the changes in the cartilage and subchondral bone. In the fat pad, *MMP13* was upregulated in the distracted joint compared to the OA control.

## Discussion

Although the clinical efficacy of joint distraction has been assessed, the underlying regenerative mechanisms behind distraction remain poorly understood. This study explores transcriptional regulation in all relevant joint tissues midway the distraction period. These unpresented results demonstrate that the regenerative response of KJD, 25 weeks after the 8-week distraction in a canine OA model,^18^ is fronted by an increased breakdown of the extracellular matrix (ECM) of the OA cartilage during the distraction phase. This is corroborated by an increased histological OARSI grade compared to the OA joint, with concomitant loss of collagen type II into the intermediate cartilage layer, and an increased expression of catabolic proteolytic genes midway distraction. At the same time, several transcriptional signals were detected compatible with cartilage regenerative responses, possibly constituting the initiation of cartilage repair activity that is seen at 25 weeks follow-up.^18^

The groove model has been shown to encompass hallmarks of progressive OA.^32,33^ In line with these previous reports, the present study revealed clear degenerative cartilage changes in the OA condition, at 14 weeks post OA induction,^17,33^ indicated by a loss of PG-rich matrix. At the transcription level, the present study demonstrated mild upregulation of matrix catabolic genes *ADAMTS5* and *MMP13*, and hypertrophy like changes in chondrocytes with increased *VEGFA* and osteocalcin (*BGLAP*), known to play a role during OA.^34^ *ACAN* and *COL2A1* showed a higher expression compared with the control joints. This is in line with the increased proteoglycan synthesis reported in cartilage samples from the groove model at 10 weeks post OA induction,^32^ and other reports of the expression of *ACAN* and *COL2A1* at early OA stages.^35-37^ This enhanced chondrocyte activity in OA cartilage is an attempt for repair considered to be ineffective, as the newly formed molecules are also lost at a higher rate, resulting in a net loss of tissue.^38^

This study demonstrates for the first time that joint distraction initiates mainly a catabolic response midway distraction, mostly concentrated in cartilage and subchondral bone as shown on histology and the coinciding transcriptional regulations. Joint distraction elicited a higher OARSI cartilage score compared to OA. This increased ECM degradation corresponded with the distinct upregulation of *MMP9*, *MMP13*, and *ADAMTS5* and further reduction of their respective proteolytic targets, *ACAN* and *COL2A1.* The latter was further corroborated by a decrease of collagen type II staining into the intermediate cartilage layer. The observed imbalance between matrix anabolism and catabolism during distraction seems to be contra-intuitive in respect of the final outcome of distraction; several human and animal studies demonstrated the improvement in ECM quantity and quality.^3-6,8^ However, from animal and human studies, it is known that an absence of normal joint loading causes a reduction in PG content, a decreased PG synthesis, and thinning of the (calcified) cartilage.^39-41^ Additionally, in rabbits, a 9-week distraction period of healthy knee joints resulted in degenerative changes in the articular cartilage similar to those in early OA.^42^ Nonetheless, Wiegant *et al*.^18^ employed the groove canine model in a similar fashion as in the present study and demonstrated 25 weeks after KJD a significant improvement of the histological OARSI grade compared to the untreated OA knee joints.

Hypothetically, the dominating catabolic stimuli during the unloading of the joint that deplete the ECM may allow for remodeling with healthy cartilaginous matrix on the long term. For example, if aggrecan molecules are enzymatically truncated, but not removed from the hyaluronic acid core in the process of OA, and with that from the matrix, that aggrecan molecule cannot be replaced, leaving an impaired aggrecan complex.^43^ Only upon further degradation is the truncated molecule removed and fully replaced. Within this context, even though matrix catabolism seems to predominate at the transcriptional and protein level halfway the distraction phase, there are some important differences in the transcriptional response of the distracted compared to the OA cartilage. These differences are discussed below and could provide insight into several suitable regenerative mechanisms by which distraction could stimulate an intrinsic cartilage repair at a later stage.

One of these proposed mechanisms is the involvement of stem cells. There is increasing evidence for the presence of chondroprogenitor cells in cartilage, even in OA.^28^ In this study, several markers were upregulated in distraction versus OA, such as *CD105*, *CD166*, *Notch-1*, *CD90*, and *CD146*, that are associated with chondroprogenitor cells.^44-48^ Whether this is because of an increased amount and/or activity of progenitor cells in the cartilage or because of the attraction and retention of mesenchymal stromal cells (MSC) from surrounding tissues remains to be further clarified. The latter has been proposed to be initiated by the intermittent fluid oscillations in the joint during distraction.^49^ Recently, it was shown that unloading of the joint by KJD resulted in a significant increase in synovial fluid MSC (SF-MSC) colony size and density.^50^ Furthermore, after 3 weeks of KJD treatment many transcriptional changes were found in these SF-MSC compared to baseline. These changes included a sustained upregulation of *ACAN* and a significant increase of the MSC chondrogenic commitment markers gremlin 1, and growth differentiation factor 5 (*GDF5*), markers associated with cartilage homeostasis and OA, among others.^50-52^ In addition, the joint environment during KJD is changed, favoring attachment of MSC.^21^

Another proposed mechanism is the effect of the periarticular bone turnover on the cartilage, as a decrease in subchondral bone sclerosis has been reported in humans and rats after KJD, which was directly associated with the reported clinical improvement.^20,23^ The present study further corroborates these findings: in the distracted subchondral bone compared to the OA control reduced transcription of the majority of the investigated matrix genes was observed together with decreased *OPG* and *RANKL*, representatives of the RANKL/RANK/OPG pathway involved in bone remodeling. Noteworthy, and in line with concepts from the rheumatoid arthritis field,^53^ *RANKL* was profoundly upregulated in the distracted cartilage and may mediate subchondral bone remodeling upon its diffusion.

Other pathways that emerged, and could provide clues for further research, were the IGF pathway, the notch pathway and the TGF/BMP pathway. TGF-β mediated signal transduction via the Smad2/3 pathway is generally thought to be a protective factor for cartilage,^24^ while an increased ALK1/ALK5 ratio, promoting Smad1/5/8 pathway signal transduction, is associated with increased *MMP13* expression, an OA hallmark.^54^ In the distracted cartilage, *ALK1* expression was upregulated compared to the OA control, corresponding with the upregulation of *MMP13* in the distracted joint. However, *PAI1*, the downstream mediator of the Smad2/3 pathway, and thereby the PAI1/ID1 ratio, was upregulated in the distracted cartilage. Furthermore, *TGFβRII* was upregulated in the tibial plateaus. This receptor has been associated with a chondroprotective role, as its expression is downregulated in human OA chondrocytes.^24^ These findings coincide with the finding of the study of Watt *et al*., which found an upregulation of TGF-β1 in the synovial fluid of human patients after 6 weeks of KJD (directly after treatment) compared to baseline.^55^ In this study, *TGF-β1* was also upregulated, though not statistically significant.

Finally, inflammatory processes during joint distraction may also be at play. *IL-6*, *IL4 receptor* (*IL4R*), *IL-10*, *IL-10R* were significant upregulated in the cartilage, and *IL-6* was 4-fold upregulated in the synovial tissue of distracted joints compared to the OA joints. In line with these findings, an upregulation of IL-6 and *CCL2*, also referred to as monocyte chemoattractant protein 1 (MCP1), was also found in the synovial fluid of human patients after the 6-week KJD treatment compared to baseline.^55^ Together with the upregulation of *IL-10*, and the *IL4-* and *10 receptor*, shown to have a chondroprotective and anti-inflammatory role,^56,57^ there seems to be involvement of multiple anabolic pathways that might initiate the reparative response generated by KJD on top of the (initial) clear catabolic activity. Upregulation of IL-10 in the blood and synovial fluid was also found in rabbits treated for 4 weeks with joint distraction and excercise.^58^ Finally, a downregulation of *IL-1β* was found in the distracted cartilage and subchondral bone compared to the OA joints. Downregulation of IL-1β was also reported by studies that investigated the effects of joint distraction in rats and rabbits,^20,58^ and also points toward a chondroprotective effect of joint distraction.

Some caution is warranted interpreting these results. This study contained only a small number of dogs per group and is therefore clearly exploratory. Dogs have been used before to study KJD,^18,59^ and provide many advantages. The anatomy of the dog knee, as well as the biochemical and histological characteristics of their cartilage and subchondral bone is similar to that of humans.^19^ In addition, canine OA progresses similar to that of humans and they do not show the spontaneous cartilage repair that is reported in rabbits.^19,60^ However, weight bearing of the limb during the distraction period could have been suboptimal as quadruped dogs are easily able to walk on 3 limbs. This could diminish the effect of the intermittent fluid changes proposed to elicit a beneficial effect on joint health.^18^ Importantly, KJD is a dynamic process and the information provided by transcriptional profiling is a static representation of a single time point without guaranteed translation to the protein level. An interesting approach for the future would be to investigate multiple follow-up time points to identify the catabolic-to-anabolic turning point in the distracted joint.

## Conclusion

This study showed for the first time that treatment of knee OA with joint distraction initiates catabolic as well as anabolic transcriptional responses. This results in a catabolic joint environment halfway during joint distraction, with aggravation of OA at the histological and transcriptional levels. This explorative study provides clues for future studies that focus on elucidating the mechanisms behind joint distraction, including the involvement of progenitor cells and the cross-talk between subchondral bone and cartilage, and the role of pro- and anti-inflammatory cytokines.

## Supplemental Material

sj-pdf-1-car-10.1177_19476035211014595 – Supplemental material for Enhanced Extracellular Matrix Breakdown Characterizes the Early Distraction Phase of Canine Knee Joint Distraction Click here for additional data file. Supplemental material, sj-pdf-1-car-10.1177_19476035211014595 for Enhanced Extracellular Matrix Breakdown Characterizes the Early Distraction Phase of Canine Knee Joint Distraction by Michelle Teunissen, Alberto Miranda Bedate, Katja Coeleveld, Frank M. Riemers, Björn P. Meij, Floris P. J. G. Lafeber, Marianna A. Tryfonidou and Simon C. Mastbergen in CARTILAGE

sj-xlsx-1-car-10.1177_19476035211014595 – Supplemental material for Enhanced Extracellular Matrix Breakdown Characterizes the Early Distraction Phase of Canine Knee Joint Distraction Click here for additional data file. Supplemental material, sj-xlsx-1-car-10.1177_19476035211014595 for Enhanced Extracellular Matrix Breakdown Characterizes the Early Distraction Phase of Canine Knee Joint Distraction by Michelle Teunissen, Alberto Miranda Bedate, Katja Coeleveld, Frank M. Riemers, Björn P. Meij, Floris P. J. G. Lafeber, Marianna A. Tryfonidou and Simon C. Mastbergen in CARTILAGE

sj-xlsx-2-car-10.1177_19476035211014595 – Supplemental material for Enhanced Extracellular Matrix Breakdown Characterizes the Early Distraction Phase of Canine Knee Joint Distraction Click here for additional data file. Supplemental material, sj-xlsx-2-car-10.1177_19476035211014595 for Enhanced Extracellular Matrix Breakdown Characterizes the Early Distraction Phase of Canine Knee Joint Distraction by Michelle Teunissen, Alberto Miranda Bedate, Katja Coeleveld, Frank M. Riemers, Björn P. Meij, Floris P. J. G. Lafeber, Marianna A. Tryfonidou and Simon C. Mastbergen in CARTILAGE

sj-xlsx-3-car-10.1177_19476035211014595 – Supplemental material for Enhanced Extracellular Matrix Breakdown Characterizes the Early Distraction Phase of Canine Knee Joint Distraction Click here for additional data file. Supplemental material, sj-xlsx-3-car-10.1177_19476035211014595 for Enhanced Extracellular Matrix Breakdown Characterizes the Early Distraction Phase of Canine Knee Joint Distraction by Michelle Teunissen, Alberto Miranda Bedate, Katja Coeleveld, Frank M. Riemers, Björn P. Meij, Floris P. J. G. Lafeber, Marianna A. Tryfonidou and Simon C. Mastbergen in CARTILAGE
