# Supplementary material for: Enhanced Extracellular Matrix Breakdown Characterizes the Early Distraction Phase of Canine Knee Joint Distraction
Source: Cartilage. 2021 May 20;13(2 Suppl):1654S–1664S. doi: 10.1177/19476035211014595 (PMC8721609; doi:10.1177/19476035211014595)
Supplement: sj-pdf-1-car-10.1177_19476035211014595 – Supplemental material for Enhanced Extracellular Matrix Breakdown Characterizes the Early Distraction Phase of Canine Knee Joint Distraction [file sj-pdf-1-car-10.1177_19476035211014595.pdf]

Supplementary Material

Animal Procedures

Upon ethical approval by the Ethics Committee on Animal Experimentation of Utrecht University (DEC no. 2013.III.08.054), skeletally mature Mixed breed dogs (n = 8 females, mean age 29±7.6 months, mean weight 23±2.8 kg) were housed in groups of pairs in indoor pens (3x4 m^2 area), and were exercised in groups on a large patio (6x8m^2 area) for at least 2 hours a day during the entire experimental period. Feeding consisted of a standard diet and water *ad libitum.*

Induction of OA The joint was approached by medial mini-arthrotomy close to and parallel with the *ligamentum patellae*. Using a Kirschner-wire (1.5-mm diameter) bent 0.4 mm from the top at 90° (ensuring that the depth of the grooves was restricted to the cartilage depth and not to the subchondral bone), ten grooves were made in the cartilage of the lateral and medial condyles with the joint in maximum flexion. Synovial tissue, joint capsule, and skin were sutured according to their anatomical layers. Burprenorphine and carprofen were used perioperatively for pain management, and carprofen alone until three days after surgery for additional pain management. Subsequently, the animals were allowed on a daily basis on the patio again. They were fully active, with subjectively normal joint loading and movement.

Joint distraction Three bone pins (3mm in diameter; Stryker®) were manually drilled into the femur, two distally (medial and lateral side) and one proximally on the craniolateral side. In addition, three bone pins were drilled into the tibia, two proximally (medial and lateral side) and one distally on the craniomedial side. External fixation frames were custom-made and adapted to the dog's anatomy. On both the tibia and the femur, a frame (5mm diameter rod) was connected to the bone-pins in a three-point fixation with use of commercially available

connectors (Delta coupling, Stryker®). Subsequently, the external fixation frames on the femur and the tibia were connected by hinges medially and laterally of the knee joint (Figure I). Distraction of the joint was carried out by extending the connecting rods and was visualized by fluoroscopy using a C-arm, while smooth motion of the joint during flexion and extension was maintained. Pain management was similar peri- and post-operatively, as described above. After 5 days of recovery, all dogs were given access to the patio again. They were active but slightly less than untreated OA controls, as observed by the animal technicians and study coordinator. Joint distraction was monitored every two weeks by radiography in a loaded position (standing), and adjustments were not necessary. During the whole experiment, no adverse events were reported.

Statistical analysis

ΔCt, and OARSI values, were statistically analyzed (*RStudio*, http://www.rstudio.com/) for the 3 comparison groups (OA vs. control, distraction vs. OA, distraction vs. control). Linear Models were employed for the analysis of variance (ANOVA). The selection of random effects for the different linear models (one per gene, bony end, and tissue) was done by an exhaustive method (*regsubsets*() function of leaps R package), considering the following variables: “donor”, “location” (medial/lateral compartment) and tibial plateaus/femoral condyles (if applicable depending on the tissue). Normality of the residuals (function *shapiro.test*() from stats R package), homoscedasticity (function *bptest*() from *lmtest* R package), independence of errors and the presence of outliers (function *durbinWatsonTest*() and *outlierTest*() from car R package, respectively) were assessed for each linear model. If any of the assumptions was not held, we performed a power transformation of the dCt values with the *lambda* coefficient as exponent (function *powerTransform*() from car R package), reassessing all the assumptions. Only for those genes in where the linear model passed all the assumptions, direct or power transformed, this model was applied. If not, an *Exact Wilcoxon-*

Mann-Whitney test, which is a permutation based non-parametric test, was used. P values were subjected to corrections for multiple testing (Benjamini-Hochberg False Discovery Rate).

Effect sizes (ES) and ES's confident intervals (95%) were calculated and provided as *Hedge's g* (normally distributed data) or *Cliff's delta* (non-normally distributed data) (Table III). To describe the ES, the rules provided by Sawilowsky S. [2] were used for *Hedge's g*: none if ES≤0.01, very small if 0.01≤ES<0.2, small if 0.2≤ES<0.5, medium if 0.5≤ES<0.8, large if 0.8≤ES<1.2, very large if 1.2≤ES<2 and huge if ES≥2; for *Cliff's delta*, the rules provided by Vargha and Delaney [3] were used: small if ES<0.28, medium if 0.28≤ES<0.43, large if 0.43≤ES<0.7, and very large if ES≥0.7. As threshold for this study, none, very small, or small ES were considered as uncertain, independently of its p-value; if p≤0.05 with medium and larger ES, if 0.05<p≤0.1 with large and larger ES and if 0.1<p≤0.2 with very large (*Cliff's delta*) or huge ES (*Hedge's g*), were considered as relevant or as substantive significant [4, 5].

A power analysis was conducted to calculate the correct number of animals and showed a minimal total sample size of 12 animals, distributed over 3 groups. For this purpose, G*Power 3.1.9.2, ANOVA: Fixed effects, omnibus, one way is employed with an alpha corrected for multiple testing, for 3 relevant comparison (control vs. OA, control vs. distraction and OA vs distraction) the alpha will be set at 0.017, and the power at 0.8. The size effect (1.6) is estimated based on the expected change based on historical data with the same animal model.

Fig. S1: Representative macroscopic and radiographic visualization of the canine joint distraction model. The external device is fixed to the canine patient by bone pins, as in the human situation.

Fig. S2: Collagen type 1 (COL1A1) and type X (COLX) immunohistochemistry.

Table Legends

Table S1: Details of the primary antibodies and the immunohistochemistry protocols employed. Mab: monoclonal antibody; **Pab**: polyclonal antibody; **Hyaluronidase**: bovine hyaluronidase (450 IU/mg, 4 mg/ml adjust to pH 5 with 0.1M HCl; **Pronase** (Roche, 11459643001): 0.1% pronase in PBS; **Pepsin** (DAKO, S3002): 5% pepsin in 0.2M HCl; **Citrate buffer**: 10mM citrate, buffer adjusted to pH 6; **PBS/BSA**: Phosphate Buffered Saline/Bovine Serum Albumin; **PBST**: Phosphate buffered saline 0.1% Tween-20; **TBS**: Tris/HCl 1M + NaCl 8.76%, adjusted to pH 7.4.

Table S2: Primers used in the RT-qPCR analysis. Primers are divided in functional groups. F; forwards, R; reverse sequence. * are genes that were additionally analyzed for cartilage.

Table S3: Effect sizes (ES), ES's confident intervals (95%, between brackets) and P values for the canine histopathology OARSI score. Provided for the macroscopic and histological assessments of each comparison (OA vs Healthy, KJD vs Healthy, and KJD vs OA) for the tibial plateaus and femoral condyles. Color label for the effect size; large ES (orange; 0.80-1.20), very large ES (blue; 1.20-2.0), and huge ES (green; ≥ 2.0).
